# Gut microbiome of mothers delivering prematurely shows reduced diversity and lower relative abundance of *Bifidobacterium* and *Streptococcus*

**DOI:** 10.1371/journal.pone.0184336

**Published:** 2017-10-25

**Authors:** Cecilie Dahl, Maggie Stanislawski, Nina Iszatt, Siddhartha Mandal, Catherine Lozupone, Jose C. Clemente, Rob Knight, Hein Stigum, Merete Eggesbø

**Affiliations:** 1 Department of Environmental Exposure and Epidemiology, Norwegian Institute of Public Health, Oslo, Norway; 2 University of Colorado School of Medicine, Colorado, United States of America; 3 Public Health Foundation of India, Gurgaon, India; 4 Department of Genetics and Genomic Sciences and Icahn Institute for Genomics & Multiscale Biology, Icahn School of Medicine at Mount Sinai, New York, United States of America; 5 Department of Medicine, Division of Clinical Immunology and Immunology Institute, Icahn School of Medicine at Mount Sinai, New York, United States of America; 6 Departments of Pediatrics and Computer Science & Engineering, University of California San Diego, San Diego, United States of America; 7 Department of Non-Communicable Diseases, Norwegian Institute of Public Health, Oslo, Norway; Colorado State University, UNITED STATES

## Abstract

**Objective:**

Preterm birth is the main reason for neonatal deaths worldwide. We investigate whether maternal gut microbiota may play a previously overlooked role.

**Methods:**

The Norwegian Microbiota Study (NoMIC) is a case control study on preterm birth (<259 days of gestation, calculated primarily based on the last menstrual period), including two consecutively born term infants per infant born prematurely. Eligible mothers were fluent in Norwegian and recruited from the maternity ward at a county hospital in Eastern Norway in the period 2002–2005. Fecal samples were collected at day 4 postpartum, and analyzed using 16S ribosomal RNA gene sequencing. We used samples from 121 mothers giving birth vaginally. Measures of alpha diversity (Shannon, Phylogenetic Diversity and Observed Operational Taxonomic Units) and microbiome composition were combined with information from the Medical Birth Registry, pregnancy journals, and questionnaires.

**Results:**

The association between maternal gut diversity and preterm delivery was examined using logistic regression. One IQR increase in Shannon diversity was significantly associated with 38% lower odds of spontaneous preterm birth, (95% confident interval (CI): 1%, 61%), and the association was stronger when adjusting for maternal age, marital status, ethnicity, parity, BMI, education, antibiotic use, pets in the household, income and smoking (48% lower odds, 95% CI: 4.2%, 72%). Mothers delivering prematurely also had lower abundance of OTUs belonging to *Bifidobacterium* and *Streptococcus*, and of the Clostridiales order.

**Conclusion:**

Analysis of maternal gut microbiota using next-generation sequencing shows that low gut diversity, with a distinct microbial composition, is associated with spontaneous preterm delivery.

## Introduction

Preterm delivery, defined as birth before 37 weeks of pregnancy, is the cause of many neonatal deaths worldwide[1], but the underlying etiology is not well understood [2, 3]. Surviving children are at risk of poor health outcomes including necrotizing enterocolitis, respiratory illnesses, sensory deficits and learning disabilities [3]. Even healthy late-preterm (34–36 week) children exhibit subtle deficits in cognitive functioning [4]. Despite advances in care, preterm births have continued to rise, partially due to increases in assisted reproductive technology (ART) treatment and multiple pregnancies [3]. Deeper insight into factors contributing to preterm delivery is needed. About half of preterm deliveries are spontaneous (i.e. not surgically or medically induced), either in the absence or presence of premature rupture of membranes [3]. Many maternal factors are associated with spontaneous preterm delivery, including previous premature birth, age and parity, low socioeconomic status, non-Caucasian origin and smoking before conception [3]. However, few previous studies have taken these factors into account when investigating spontaneous preterm delivery and microbiota, which may have biased the results. Intrauterine inflammation due to vaginal infections or imbalance in the vaginal flora, with potential pathogens ascending into a near sterile intrauterine environment, is the cause of many spontaneous preterm deliveries [5–7]. One example is bacterial vaginosis, an imbalance often characterized by overgrowth of *Gardnerella* and/or *Ureaplasma* with too few *Lactobacilli* and a high vaginal diversity [8, 9]. Spontaneous preterm delivery has also been linked to infections outside the vagina, e.g. periodontitis, upper and lower urinary tract infections, and inflammatory bowel disease (IBD) [10]. The risk of spontaneous preterm delivery is highest in IBD mothers with flaring disease during pregnancy, indicating a strong relation with inflammation [10].

The gut microbiota plays complex roles in health and disease, including but not limited to immunomodulation in the gut and balancing uptake of nutrients and resistance against pathogens [11–13]. Most gut bacteria co-habit with the host in a symbiotic relationship [12], and next-generation DNA sequencing has made it possible to identify their association with health outcomes. A rich and diverse microbial composition in the gut indicates increased robustness and strength against pathogen attack [12, 13]; whereas low gut diversity is found in diseases associated with a heightened inflammatory state, such as IBD and obesity [12]. Low diversity microbiomes from pregnant women induce inflammatory markers when transplanted into germ free mice, indicating that low gut diversity may be one underlying cause of increased inflammation [14]. In the absence of sudden influences, such as disease or long-term change in diet, the microbiome remains stable throughout life [15]. Whether the gut microbiome changes during pregnancy is not clear. A decrease in gut alpha diversity and an increase in Proteobacteria and Actinobacteria have been seen when comparing the third trimester to the first [14]. However, recent studies report the gut microbiome in pregnancy to be overall stable, particularly throughout the perinatal period [8, 16].

Although preterm delivery is associated with inflammatory diseases in the gut, the role of the maternal gut microbiome in preterm delivery has not been fully studied. Our primary aim is to investigate whether gut microbial diversity in the mother is associated with spontaneous preterm delivery using next-generation sequencing, while taking into account potential confounding factors such as antibiotic use during pregnancy. Secondly, we use random forest classification to identify taxa that may be differently abundant in the maternal gut of spontaneous preterm compared with term delivery. We hypothesize that premature birth will be associated with lower overall diversity and differences in specific taxa in the maternal gut microbiome postpartum when compared to full term births.

## Materials and methods

### Study cohort

The Norwegian Microbiota Study (NoMIC) was established to study the composition of mother and infant gut microbiota, and subsequent health outcomes. Participating mothers were recruited in the maternity ward at a county hospital (Sykehuset Østfold) from 2002–2005. We samples cases and controls by convenience sampling; for each infant born prematurely (case) we sampled two consecutively born term infants (controls).

Mothers who were fluent in Norwegian and resided in Eastern Norway were eligible to participate. Questionnaires and container for fecal sample were distributed to the participants at the maternity ward. Mothers were asked to collect and freeze one sample from herself at postpartum day 4. Study personnel retrieved samples and kept them frozen during transport. Samples were stored at -20°C at the Biobank of the Norwegian Institute of Public Health upon arrival. More details on the study population can be found in Eggesbø et al 2011[17]. For the current study, the mothers’ samples taken at day 4 postpartum were used to compare diversity in preterm and term mothers. High quality Illumina results (>1,000 OTUs) from sequencing the V4 16S rRNA region were available in 183 mothers, but only 121 mothers (102 term/19 preterm) who had delivered vaginally, and who had not reported antibiotic use on or after the day of delivery were used in the main analysis.

### Ethical approvals

The NoMIC study was approved by the Norwegian Data Protection Authority and the Regional Committees for Medical and Health Research Ethics. Written, informed consent was obtained from each participant prior to study start.

### Outcome

Gestational age, obtained from the Medical Birth Registry (MBR), was primarily calculated based on the last menstrual period (LMP). Ultrasound estimates were only used if the discrepancy between LMP and ultrasound exceeded 14 days [18]. Gestational age was dichotomized into term/preterm status where preterm was defined as birth before 259 days (37 weeks). Only vaginal deliveries, as an approximation of spontaneous deliveries, were included in the main analysis; however gut diversity in mothers of caesarian sections were included in a sensitivity analysis to explore possibilities of reverse causation (S1 File).

### Exposure

For further details on DNA purification of fecal samples, PCR, sequencing and data-processing, see Supporting information (S1 File). Gut diversity at 4 days postpartum was used as a proxy for diversity at the end of pregnancy. Mothers who took antibiotics on or after the day of delivery were excluded (n = 22) (S1 Fig). Between communities diversity (beta diversity) was calculated using unweighted unifrac and visualized with Principal Coordinate Analysis (PCoA). Unifrac evaluates microbiota similarity based on shared evolutionary history of bacterial taxa [19], but does not control for subject covariate information. Alpha diversity is a measure of diversity within a subject [20], which enables comparisons between mothers. We focused on three alpha diversity measures: Shannon, Phylogenetic Diversity (PD) and Observed OTUs (Operational Taxonomic Units). Shannon takes into account the total number of species and their relative abundances. PD is based on the proportion of branch length in a phylogenetic tree that leads to different organisms, whereas observed OTUs is the number of unique OTUs found in the sample. All measures were calculated in the Quantitative Insights Into Microbial Ecology (QIIME) version 1.7.0 [21].

### Covariates

We identified potential covariates through literature search and selected them based on Directed Acyclic Graphs (DAGs, www.daggity.net, Supplemental Material (S2 Fig). Covariates included were: maternal age, marital status, ethnicity, parity, BMI, education, antibiotic use in pregnancy, household pets, income and smoking. From the questionnaires we obtained maternal age at the start of pregnancy (years), marital status while pregnant (married/not married including living together), ethnicity (Norwegian, i.e. both the mother and her parents born in Norway/not Norwegian), parity (none vs. one or more previous children), household pets (yes/no) and antibiotic use during pregnancy. Antibiotic use was reported early in pregnancy, 1 month before delivery, the week before delivery and/or the day before delivery, and collapsed into “any antibiotics used during pregnancy (yes/no)”.Body mass index (BMI) (kg/m^2^) at the beginning of pregnancy was calculated from reported weight and height in the pregnancy journal. Length of education was given in 3 categories (< 12 years/12 years/≥ 12 years). The MBR provided data on income (Euros) and smoking at the beginning of pregnancy (yes, including occasionally/no).

### Statistical analyses

Wilcoxon rank sum-test was used to infer differences according to covariates. We used simple and multiple logistic regression models (odds ratios [OR] with 95% Confidence Intervals [CI]) to detect association between maternal gut alpha diversity and spontaneous preterm delivery. Shannon, Phylogenetic Diversity and Observed OTUs, along with maternal age, BMI and income were treated as continuous variables throughout the analysis; the other variables were binary or categorical. The number of missing values ranged from 1 to 33 (S1 Table), and were replaced using multiple imputations (MI). Fifteen datasets were imputed using predictive mean matching in STATA 14 based on all the variables in the full model, in addition to weight and height, asthma, smoking, birthweight of the child and mode of delivery (caesarian or vaginal), a total of 64 complete cases. The adjusted analysis using MI were compared to the results from a complete case analysis. In a sensitivity analysis we included mothers with a spontaneous start of labor with premature rupture of membranes (PROM), even if they had a subsequent caesarian delivery. In the same sensitivity analysis we also excluded deliveries that were medically induced (i.e. by amniocentesis, prostaglandin and oxytocine), giving a total of 111 deliveries, 94 term, 17 preterm (S1 Fig). For further details on MI, quality control and sensitivity, see Supporting information (S2 File). OTUs important for differentiating term/preterm status were identified using random forests. The random forest approach ranks factors based on their ability to discriminate between the groups, while taking into account the complex interrelationships in high dimensional data [22]. We included only the 508 OTUs that were nonzero in at least 5% of the samples and had a minimum relative abundance of at least 0.25. The ranking of OTUs according to mean decrease in accuracy were obtained from the random forest algorithm using default parameters in the R package (R 3.2.0) ‘randomForest’ (with ntree = 10,001) [23]. Down sampling was used to account for the difference in sample size in the term and preterm groups. To estimate the minimal number of top ranking OTUs required for accurate prediction of status, the rfcv function (also in the ‘randomForest’ package) was applied. Based on these results, the top 25 OTUs were passed to the function varSelfRF with default parameters [24]. This function uses the out of bag error as minimization criterion to successively eliminate the least important variables from the random forest and selected 8 OTUs. The selected OTUs were also used in a random forest with down sampling in order to quantify the out of bag error and classification error. The random forest had an out of bag error rate of 16.5%; the classification error was 13.7% for term and 31.5% for preterm. Since the varSelfRF function does not use down sampling, the OTUs selected by this function were compared to the results of a random forest of the same top 25 OTUs with down sampling. The results were very similar with all the 8 OTUs identified by the varSelfRF function being among the top 9 most important OTUs according to either mean decrease in accuracy or Gini. The selected OTUs, as well as phylum and family level taxa, were further compared between term/preterm groups using Wilcoxon ranksum-tests, applying the Benjamini-Hochberg procedure for multiple comparisons correction (q<0.05 deemed significant). An additional nucleotide search was performed through NCBI BLAST (http://blast.ncbi.nlm.nih.gov/Blast.cgi).

## Results

The median (range) gestation of the preterm group was 237(81) days. Table 1 shows that there were no large differences between the mothers sampled in the current study, compared with all mothers in NoMIC, and with the general population of birth giving mothers, except for a somewhat higher proportion of non-smokers in the sampled group.Table 2 compares spontaneous preterm delivery/term deliveries across covariates. This study had 36% more preterm mothers with household pets, and 15% more had taken antibiotics in the week before giving birth.

**Table 1:** Mothers delivering vaginally in NoMIC compared with other NoMIC participants and the general population of birth-giving mothers in Norway (from the Medical Birth Registry).

Covariate		Vaginal deliveries in NoMIC (N = 121) ^a^	NoMIC (N = 281) ^a^	General population (N = 126,182) ^b^
**Gestational age (days, median)**		282	277	282
**Birthweight (gram, median)**		3,450	3,330	3,570
**Child sex (%)**				
	**Female**	51.2	46.9	48.7
	**Male**	48.8	53.0	51.2
**Maternal age (years, median)**		30	30	29
**Parity (%)**				
	**First child**	43.8	43.4	43.8
	**Second child**	38.8	37.4	35.3
	**Third child or more**	17.4	19.2	20.8
**Smoking ^c^ (%)**				
	**No**	92.5	85.7	86.8
	**Occasional**	2.2	2.7	1.1
	**Daily**	5.4	11.6	12.1

^a^ N varies depending on the covariate (see S1 Table)

^b^ Information on the general population of recent mothers was obtained through the Norwegian Medical Birth Registry (which records all births in Norway). Mothers who had given birth between 2001 and 2003 were selected and their characteristics compared to the NoMIC participants.

^c^ Smoking at the beginning of pregnancy

**Table 2:** Mothers of preterm and term vaginal deliveries (N = 121) compared across covariates in the NoMIC study.

Covariate		Term N = 102	Preterm N = 19	p-value^a^
**Mother’s age (median years)**		29.5	31	0.85
**Income (median Euro)**		75 k	64 k	0.26
**Maternal BMI (median kg/m^2^)^b^**		22.7	23.6	0.97
**Marital status(%)^c^**				
	**Living together/single**	63	47	
	**Married**	36	53	0.20
**Parity (%)**				
	**First child**	42	53	
	**Second child or more**	58	47	0.46
**Education (%)**				
	**≤ 12 years**	4	12	
	**12 years**	16	12	
	**>12 years**	80	77	0.33
**Ethnicity (%)**				
	**Other**	13	11	
	**Norwegian**	87	88	1.00
**Smoking (%)^d^**				
	**No**	94	86	
	**Yes**	6	14	0.28
**Mother has pets (%)**				
	**No**	59	23	
	**Yes**	41	77	0.03
**Antibiotics early in pregnancy (%)**				
	**No**	88	82	
	**Yes**	12	18	0.46
**Antibiotics the month before delivery (%)**				
	**No**	94	94	
	**Yes**	6	6	1.00
**Antibiotics the week before delivery (%)^e^**				
	**No**	97	82	
	**Yes**	3	18	0.04

^a^ Wilcoxon ranksum test (continuous covariate) or Fisher exact test (categorical covariate)

^b^ BMI at start of pregnancy

^c^ Marital status during pregnancy

^d^ Smoking at the beginning of pregnancy

^e^ 9 women (5 preterm and 4 term) reported antibiotic use the week before delivery. Reasons for antibiotic use: Urinary tract infection (N = 2 preterm, N = 2 term), respiratory infection (N = 1 preterm, N = 2 term), premature rupture of membranes (N = 2 preterm), infection of unknown reason (N = 1 preterm). One woman reported 2 reasons

### Diversity

PCoA showed no difference in the maternal beta diversity of preterm vs. full-term deliveries (S3 Fig). Table 3 gives odds ratios per one unit increase in alpha diversity, unadjusted and adjusted for covariate information. Median Shannon diversity in the mothers was 5.8, with an interquartile range (IQR) of 0.83 (Table 3). One IQR increase in Shannon diversity was associated 38% (95%CI: 1%,61%) lower odds of having a spontaneous preterm birth. The association between preterm and alpha diversity was stronger in the adjusted analysis after multiple imputations of missing values, 48% (95%CI: 4.2%,72%) lower odds of preterm delivery per IQR increase in Shannon. A significant association was also found with PD and Observed OTUs (Table 3). The results from the adjusted analysis with only complete cases (Table 3) were different from the results where all 121 mothers were included by imputing values for missing information; however, other sensitivity analyses did not change the results.

**Table 3:** Association between maternal gut diversity and spontaneous preterm delivery (N = 121) from the NoMIC study.

Alpha diversity ^a^	Median	IQR^b^	OR (95% CI), unadjusted^c^	OR (95% CI), adjusted^d^	OR (95% CI) sensitivity^e^	OR (95% CI) complete case^f^
**Shannon**	5.78	0.83	0.56 (0.32, 0.99)*	0.45 (0.22, 0.95)*	0.48 (0.23, 0.99)*	1.06 (0.13, 8.7)
**Phylogenetic Diversity (PD)**	14.08	4.41	0.86 (0.73, 1.00)*	0.81 (0.66, 0.98)*	0.79 (0.64, 0.98)*	0.95 (0.63, 1.4)
**Observed OTUs**	998	232	0.99 (0.99, 1.00)*	0.99 (0.99, 1.00)*	0.99 (0.99, 1.00)*	1.0 (0.99, 1.0)

p-value

*<0.05.

^**a**^ Due to the different scales of the metrics, the ORs are different.

^**b**^ Interquartile range: 75th-25th percentile

^**c**^ Unadjusted Odds Ratios per 1-unit change in alpha diversity with 95% Confidence Intervals (N preterm = 19, N term = 102)

^**d**^ Odds Ratios per 1-unit change in alpha diversity with 95% Confidence Intervals, adjusted for: maternal age, ethnicity, parity, marital status, BMI, education, income, antibiotic use, and pets in the household (N preterm = 19, N term = 102)

^**e**^ Odds Ratios per 1-unit change in alpha diversity with 95% Confidence Intervals. Sensitivity analysis: Including PROM and excluding medically (i.e. amniocentesis, prostaglandin and oxytocin) induced deliveries (N preterm = 17, N term = 94), adjusted for covariates in ^**c**^

^**f**^ Odds Ratios per 1-unit change in alpha diversity with 95% Confidence Intervals, complete case analysis (not multiple imputed), adjusted for covariates in ^**c**^ (N preterm = 7, N term = 57)

### Taxonomic differences

**Fig 1:**
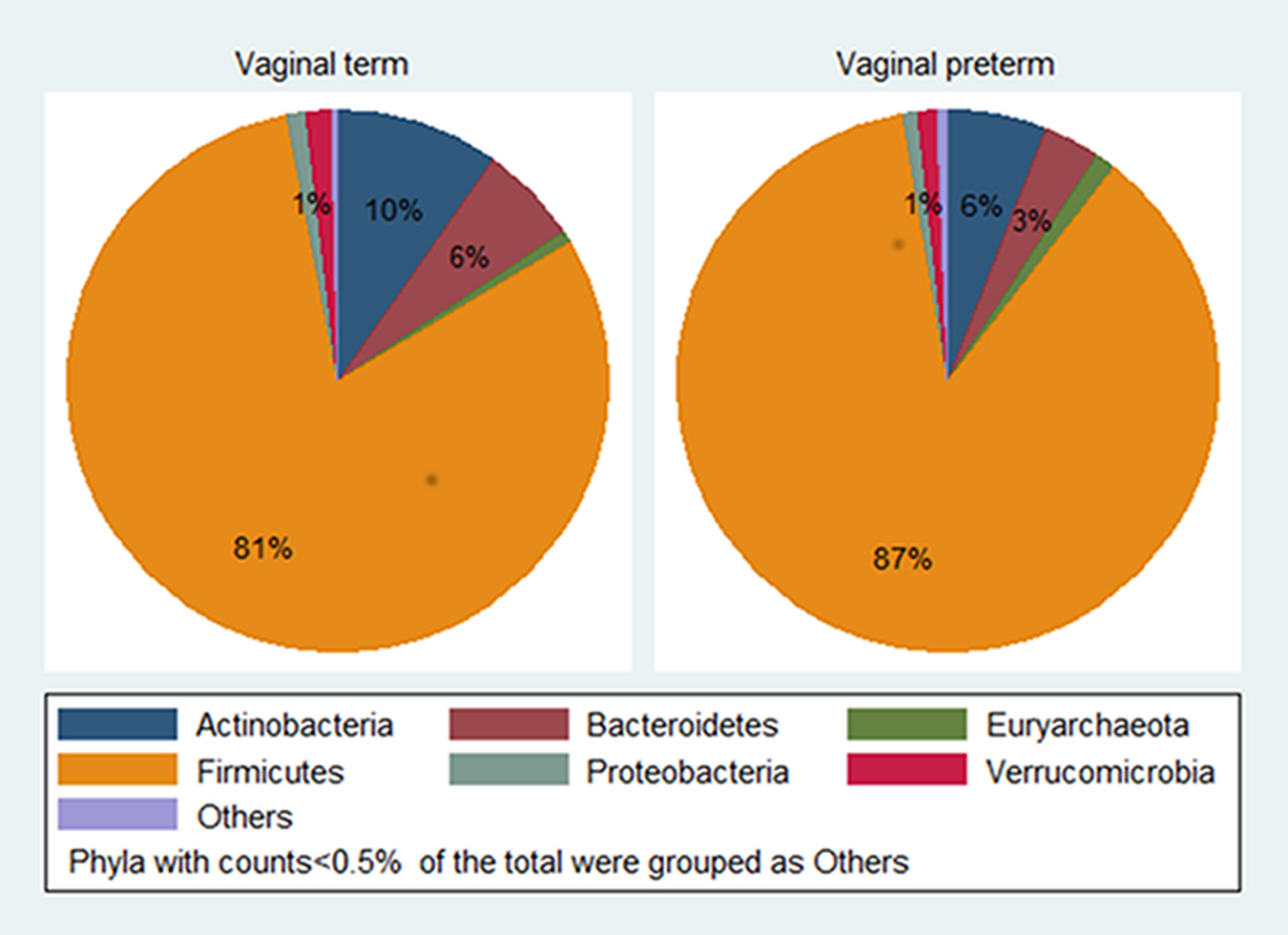
Phylum abundance in vaginal term (0) and vaginal preterm (1) deliveries, 121 mothers in the Norwegian Microbiota Study (NoMIC).

**Fig 2:**
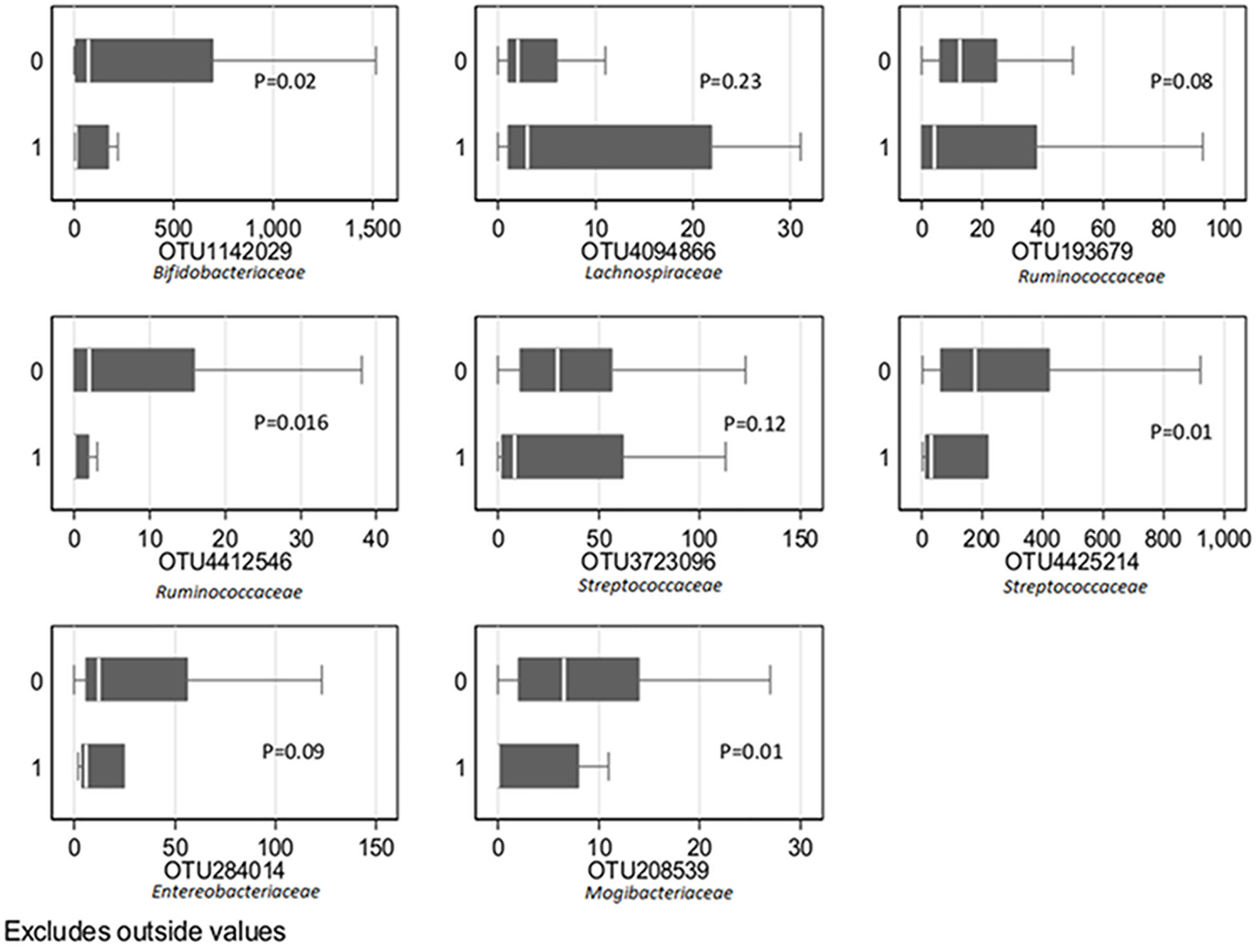
The most important OTUs in maternal gut predicting preterm deliveries. Vaginal term (0) and vaginal preterm (1) deliveries, 121 mothers in the Norwegian Microbiota Study (NoMIC). P-values from Wilcoxon rank-sum test.

According to Wilcoxon rank-sum tests, women delivering prematurely had more Firmicutes (p = 0.04), but less Actinobacteria (p = 0.01) than women delivering at term (Fig 1). Abundance of 19 families was compared (S4 Fig). A lower median abundance of the families *Streptococcaceae* and *Bifidobacteriaceae* were found in mothers of preterm compared to term deliveries (S4 Fig), but differences were not significant when applying correction for multiple-testing (q = 0.19 for both tests). Random forest results for the top 25 OTUs are summarized in Table 4. Eight OTUs were selected for further scrutiny (Fig 2), and after correction for multiple-testing, 4 OTUs were found to have a significantly lower abundance in mothers of preterm deliveries compared to term (q = 0.04, Wilcoxon rank-sum test): OTU1142029 (NCBI BLAST: *B*. *tsurumiense*), OTU4425214 (NCBI BLAST: *S*.*vestibularis* or *S*. *salivarius*), OTU4412546 (*Ruminococcaceae*, *Clostridium cluster IV*) and OTU208539 (*Mogibacteriaceae*, *Clostridium Family XIII Incertae Sedis*).

**Table 4:** Operational Taxonomic Units (OTUs) predictive of preterm delivery. Random forest analysis of 121 women giving birth vaginally in the NoMIC study.

Mean decrease accuracy^a^	Preterm median abundance^b^	OTU ID	Phylum	Class	Order	Family	*Genus*	*Species*
0,004668	low	4412546*	Firmicutes	Clostridia	Clostridiales	*Ruminococcaceae*	*-*	*-*
0,003563	low	208539*	Firmicutes	Clostridia	Clostridiales	*Mogibacteriaceae*	*-*	*-*
0,002974	low	681370	Actinobacteria	Actinobacteria	Bifidobacteriales	*Bifidobacteriaceae*	*Bifidobacterium*	*B*. *pseudolongum*
0,002699	low	193679	Firmicutes	Clostridia	Clostridiales	*Ruminococcaceae*	*-*	*-*
0,002530	high	4094866	Firmicutes	Clostridia	Clostridiales	*Lachnospiraceae*	*-*	*-*
0,002457	low	1142029*	Actinobacteria	Actinobacteria	Bifidobacteriales	*Bifidobacteriaceae*	*Bifidobacterium*	*-*
0,002124	low	48084	Firmicutes	Clostridia	Clostridiales	*Ruminococcaceae*	*-*	*-*
0,001694	No difference	4469302	Firmicutes	Clostridia	Clostridiales	*Lachnospiraceae*	*-*	*-*
0,001297	low	4306262	Verrucomicrobia	Verrucomicrobiae	Verrucomicrobiales	*Verrucomicrobiaceae*	*Akkermansia*	*A*. *muciniphila*
0,001209	low	12574	Actinobacteria	Actinobacteria	Actinomycetales	*Actinomycetaceae*	*Actinomyces*	*-*
0,001166	low	4344207	Firmicutes	Bacilli	Lactobacillales	*Streptococcaceae*	*Streptococcus*	*S*. *anginosus*
0,001160	low	1803030	Firmicutes	Clostridia	Clostridiales	*Lachnospiraceae*	*Coprococcus*	*-*
0,00112	low	4426051	Firmicutes	Clostridia	Clostridiales	*Lachnospiraceae*	*Ruminococcus*	*R*. *gnavus*
0,001775	low	4425214*; 3723096; 3166216	Firmicutes	Bacilli	Lactobacillales	*Streptococcaceae*	*Streptococcus*	*-*
0,000892	low	230232	Firmicutes	Clostridia	Clostridiales	*Lachnospiraceae*	*Dorea*	*-*
0,000805	No difference	851938	Firmicutes	Erysipelotrichi	Erysipelotrichaceae	*Erysipelotrichaceae*	*Solobacterium (Bulledia)*	*Solobacterium moorei (B*. *moorei)*
0,000787	low	176954	Firmicutes	Clostridia	Clostridiales	*Lachnospiraceae*	*-*	*-*
0,000656	low	197367	Bacteroidetes	Bacteroidia	Bacteroidales	*Bacteroidaceae*	*Bacteroides*	*-*
0,000584	low	187816	Firmicutes	Clostridia	Clostridiales	*Unkown*	*-*	*-*
0,000485	low	359872	Proteobacteria	Deltaproteobacteria	Desulfovibrionales	*Desulfovibrionaceae*	*Bilophila*	*-*
0,000483	low	4474760	Firmicutes	Clostridia	Clostridiales	*Ruminococcaceae*	*Oscillospira*	*-*
1,66E-06	low	284014	Proteobacteria	Gammaproteobacteria	Enterobacteriales	*Enterobacteriaceae*	*-*	*-*
-5,54E-05	No difference	1102370	Firmicutes	Clostridia	Clostridiales	*Ruminococcaceae*	*-*	*-*

## Discussion

In this case-control study of 121 mothers with vaginal deliveries, mothers giving birth prematurely were found to have lower alpha diversity in the gut, and the association was stronger when adjusting for potential confounders such as antibiotic use during pregnancy. The spontaneous preterm delivery mothers also had significantly lower abundance of OTUs belonging to the genera *Bifidobacterium* and *Streptococcus*, and to families in the Clostridiales order.

To our knowledge, this is the first study comparing bacterial gut diversity in mothers of preterm and term deliveries using next-generation sequencing, as well as taking into account maternal characteristics that could confound the association. Just one small previous study has investigated abundance and preterm delivery. Shiozaki et al [25] compared the gut microbiota in 10 women with preterm delivery using Terminal Restriction Fragment Length Polymorphism to women with preterm labor with term delivery (n = 11) and term deliveries without preterm labor (n = 20). Significantly lower levels of *Clostridium subcluster XVIII*, *Clostridium cluster IV* and *Clostridium subcluster XIVa* were found in the mothers of preterm birth compared to non-preterm labor (term) group, which agrees with our findings of lower abundance of several OTUs from the Clostridiales order. Contrary to our results, Shiozaki et al [25] reported a lower abundance of *Bacteroides* and a higher abundance of Lactobacillales in spontaneous preterm delivery. One OTU from *Bacteroides* was above the prevalence threshold and selected by the first round of random forest in our study; however the abundance was not significantly different between mothers of preterm and term deliveries. Additionally, a lower abundance of *Streptococcus* (Lactobacillales) was observed in spontaneous preterm delivery, not a higher as reported by Shiozaki et al [25].

We found a low bacterial diversity in women delivering prematurely, compared to women delivering at term. Low bacterial diversity in the gut of pregnant women has been shown to induce metabolic syndrome, with significantly higher markers of inflammation, in recipient mouse models [14]. An immune activation through inflammation could lead to increased production of prostaglandin, promoting uterine contractions, which along with ripening of the cervix and degradation of the fetal membranes may cause preterm birth [3].

Our most significant finding of taxonomic differences was the lower abundance of *Streptococcus and Bifidobacterium* in mothers with spontaneous preterm delivery. Koren et al [14] observed an increase in maternal gut *Streptococcus* between the first and the third trimester of pregnancy, and in postpartum samples of term mothers, hypothesizing that this enrichment may serve to educate the developing immune system in children. However, many members of *Streptococcus* are commensal, and both *Streptococcus* and *Bifidobacterium* are major producers of lactic acid, and also the short-chain fatty acid acetate[26], which suppress growth of pathogens such *as E*.*coli* across the epithelium. They also reduce inflammation in the gut in cooperation with the host’s immune system. The significant OTU classified as *Streptococcus* in the current study was identified to be closely related to *S*.*vestibularis* and *S*. *salivarius*. Not much is known about *S*.*vestibularis*, but some strains of *S*. *salivarius* produce bacteriocins that may have probiotic effects [27]. A possible association between low *Bifidobacterium* and *Streptococcus* in maternal gut and spontaneous preterm delivery has not before been reported.

Low relative abundance of OTUs from the Clostridiales order was associated with preterm delivery. The *Ruminococcaceae* are active producers of the short-chained fatty acid butyrate [28]. Higher levels of *Ruminococcaceae* may contribute in promoting Treg cell accumulation, leading to better resistance to allergy and intestinal inflammation in mouse models [29, 30], and lower levels have been reported in patients with IBD compared to healthy controls [30]. The taxonomic classification of *Mogibacteriaceae* is relatively recent [31, 32]. Some species of *Mogibacterium* have been reported to be enriched in the gut mucosa of colon cancer patients[32], and have been found in patients with periodontitis[31], but their role in pregnancy outcomes is unknown.

The case-control design with oversampling of preterms provides better statistical power, enabling restriction to spontaneous births only. Combining data from biological samples, sequenced by state of the art techniques, with rich information from questionnaires and registries, gave the possibility to control for important maternal factors such as antibiotic use during pregnancy, often lacking in other studies. In particular, reported antibiotic use one week prior to delivery, along with having pets in the household were reported to be higher in women giving spontaneous preterm birth. This may signify an underlying inflammation, as both antibiotic use and household pets are associated with infections, such as toxoplasmosis [33]. Preterm delivery mothers were less likely to complete and/or return questionnaires, which means that responses were missing more often in the preterm than in the term group. Given that the distributions of the imputed and observed values were quite similar; it could indicate that the MI analysis reduced selection bias in the current study, by correcting for the non-response among women with preterm delivery.

There are limitations, however. Participants were recruited post-delivery and child outcomes could have affected participation. We lacked information from mothers who lost their child due to extreme prematurity. The percentage of preterm deliveries in Norway is low (6%), which is why we chose to oversample mothers with preterm delivery. Still, only 19 preterm deliveries could be categorized as vaginal, with only 17 being spontaneous. With such a small case-group there is always the possibility of some individuals having a large influence on the results, but the application of Pregibon Delta-Beta influence statistic decreases potential bias from any extreme values. There is also reduced statistical power in a small study. The current study had only 35% power to detect a difference in mean values between the preterm and full term groups, still, we found a clear association between low maternal diversity and spontaneous preterm delivery. We collected fecal samples at 4 days postpartum, which is an imperfect measure of antenatal gut diversity. Our rationale for doing this was largely practical; the mothers were enrolled post-delivery based on their preterm/term status, which made sample collection before enrollment impossible. To reduce measurement error in exposure we removed mothers that reportedly had been given antibiotics on or after labor-day. A total of 22 women were removed, and the reasons for antibiotic use are listed in S2 Table. Only two of the women reported antibiotic use due to Group B *Streptococcus* (GBS), making it unlikely that our estimate of low *Streptococcus* in the preterm group was due to exclusion of women with GBS infection. It is not common practice to screen for GBS, or to give antibiotics to women with GBS in Norway, unless there are other risk factors present. The women excluded due to antibiotic use on or after the day of labor were included in a sensitivity analysis, and the strength of association between low diversity and premature delivery remained unchanged. Reliability of recall could be a problem with self-report. In the current study, data on antibiotic use in pregnancy were collected within one month after delivery, giving only a short period of recollection. Also, specifics about the antibiotic(s) were asked, making over-reporting unlikely. Gut diversity may change across pregnancy due to increasing gestational age [14], therefore our findings could simply be a result of a short gestation period in preterm mothers, i.e. reverse causation. To assess this we compared median gut diversity in mothers delivering prematurely **by caesarian section** to mothers delivering at term in NoMIC. The preterm caesarian mothers did not have lower diversity than term mothers, even with shorter gestation (S5 Fig), making reverse causation unlikely. Unfortunately, we did not have data on nutritional intake in pregnancy. Intake of probiotic foods has previously been found to influence spontaneous preterm birth in the Norwegian Mother and Child Cohort [34], and more studies are needed to determine whether this could affect the association between gut diversity and preterm birth.

## Conclusion

In this state-of-the-art study on the maternal gut microbiome’s role in preterm delivery, we report an association between low bacterial diversity and increased risk of spontaneous preterm delivery. The association was stronger when controlling for all known potential maternal confounders such as age, antibiotic use during pregnancy, ethnicity, body mass index, smoking at the beginning of pregnancy and education. Mothers giving birth prematurely had a lower relative abundance of OTUs belonging to the *Bifidobacterium* and *Streptococcus*, and to the Clostridiales order. The low gut diversity, along with the distinct microbial composition may make some women prone to increased inflammation in pregnancy, possibly leading to a higher risk of delivering prematurely. If the proposed associations are causal, new therapies such as dietary modification, supplementation with pre-, pro-, post- and/or synbiotics could aid in the prevention of preterm birth. However, this study was conducted at only one time point in mothers from one hospital, and in a country where the prevalence of preterm delivery is relatively low. Larger prospective studies with repeated gut diversity measurements during pregnancy are therefore needed to confirm our findings, and to determine whether specific microbes convey protection or risk.

## Supporting information

S1 File Supporting information: Materials and methods. (DOCX)

S2 File Supporting information: Quality control and sensitivity. (DOCX)

S1 Table Summary of variables with missing values. Complete case (CC) versus Multiple Imputed (MI) per variable. In total 64 complete cases. NoMIC study.
(DOCX)

S2 Table Reported reasons for antibiotic use in the 22 women excluded due to antibiotic use on or after the day of labor. (DOCX)

S1 Fig Flowchart for subject inclusion in main analysis; Flowchart for subject inclusion in sensitivity analysis. (DOCX)

S2 Fig Directed Acyclic Graph (DAG) for selection of covariates. (DOCX)

S3 Fig Between communities gut diversity (beta diversity) in unweighted unifrac, visualized with Principal Coordinate Analysis (PCoA). (DOCX)

S4 Fig Box plot comparisons of bacterial families with abundance >0.5% of the total. Data from 121 mothers, vaginal term delivery (0) and vaginal preterm delivery (1) in the Norwegian Microbiota Study (NoMIC).
(DOCX)

S5 Fig Gut diversity (measured by Shannon) in mothers of 4 delivery types: Vaginal term (N = 102), C-section term (N = 19), C-section preterm (N = 21) and vaginal preterm (N = 19). Mothers reporting antibiotic use during or after delivery were excluded.
(DOCX)

## Abbreviations

BMI: Body Mass Index
DAG: Directed Acyclic Graph, or causal graph
GBS: Group B *Streptococcus*
IBD: Inflammatory Bowel Disease
LMP: Last Menstrual Period
MBR: Medical Birth Registry
NoMIC: The Norwegian Microbiota Study
Shannon: Shannon diversity index, a calculated measure of gut alpha diversity
PCoA: Principal coordinate analysis
PD whole tree: Phylogenetic whole tree, a calculated measure of gut alpha diversity
Observed OTUs: Number of observed operational Taxonomic Units, a calculated measure of gut alpha diversity
OTU: Operational Taxonomic Unit, gene sequence corresponding to one specimen of bacteria
QIIME: Quantitative Insights Into Microbial Ecology
