# Supplementary material for: Gut microbiome of mothers delivering prematurely shows reduced diversity and lower relative abundance of Bifidobacterium and Streptococcus
Source: PLoS One. 2017 Oct 25;12(10):e0184336. doi: 10.1371/journal.pone.0184336 (PMC5656300; doi:10.1371/journal.pone.0184336)
Supplement: S1 File — (DOCX) [file pone.0184336.s001.docx]

**Supporting information**

**Materials and methods**

### Automated DNA purification of fecal samples

Feces were prepared for analysis by adding one milliliter Solution 1 (50 mM glucose, 25 mM Tris–HCl pH 8.0, and 10 mM EDTA pH 8.0) per 0.2 g faeces. The samples were mixed by vortexing and left for 30-60 min. on ice before 400 µl of the supernatant was diluted 1:2 in 4 M guanidinium thiocyanate (GTC). Five hundred microliters of sample were transferred to a sterile FastPrep®-tube (Qbiogene Inc., Carlsbad, CA, USA) containing 250 mg glass beads (106 microns and finer, Sigma-Aldrich, Steinheim, Germany), and samples were homogenized for 40 seconds in FastPrep® Instrument (Qbiogene). Wells in a 96-well Greiner U-plate (Greiner bio-one, Frickenhausen, Germany) were filled with 170 μl sample and 10 μl Silica particles (Merck, Darmstadt, Germany) and transferred to a Biomek® 2000 Workstation (Beckman Coulter, Fullerton, CA, USA). One percent Sarkosyl was added, and the plate was incubated at 65 °C for 10 min. and at room temperature for 10 min. The supernatant was removed, and the paramagnetic beads were washed twice with 50% ethanol. DNA was eluted from the silica particles by suspension of the particles in 100 μl Buffer C (1 mM EDTA pH 8.0, 10 mM Tris–HCl pH 8.0) at 65 °C for 30 min. The adequacy of the automated DNA extraction procedure was evaluated by repeating the DNA extraction in 20 samples using the modified MoBio 96-well manual extraction method adopted by the Earth Microbiome Project. [1] The samples gave very similar results regardless of DNA extraction method used.

### PCR

1 ul DNA extracted from fecal samples was amplified by PCR reactions by 16S rRNA specific primers (515F-806R) (http://www.earthmicrobiome.org/emp-standard-protocols/16s/). All reactions were set up as 25ul samples in 96 well Thermo-fast 96, low profile, 0,2ml, non-skirted PCR plates (ABgene Thermo scientific, UK) with Cas1200 Corbett robot (Qiagen). 10ul HotMastermix enzyme (5PRIME GmbH, Germany), 0,2uM forward-/ reverse primers (ILHS_515fa/ IL_806rcbc) and 13ul PCR grade water (Qiagen) were used.

### Sequencing and data processing

Sequencing of the V4 region of the 16S rRNA gene using the Illumina HiSeq instrument resulted in a total of 271 samples after demultiplexing (sorting by sample-specific nucleotide barcodes). Of these, 88 samples had to be discarded due to low quality (<1000 sequences). The remaining 183 had a median of 183,286 sequences per sample (minimum of 46,868). To control for variation in sequencing effort, the data were rarified at a depth of 45,000, which did not lead to any further exclusion of samples. OTUs were formed using a closed-reference OTU picking procedurein QIIME to cluster (at 97% sequence similarity) and mapped against the Greengenes 13-8 reference set of 16S rRNA gene sequences .All OTUs were assigned to bacterial taxa using the UCLUST consensus taxonomy assigner. Data processing was performed in the Quantitative Insights Into Microbial Ecology (QIIME) pipeline version 1.7.0. [2]

### Multiple imputations (MI)

Fifteen datasets were imputed using predictive mean matching in STATA 14 based on all the variables in the full model, in addition to weight and height, asthma, smoking, birthweight of the child and mode of delivery (caesarian or vaginal), a total of 64 complete cases. To reach convergence, we had to add gestational age as a full information-predictor, singly imputing the one missing value with the median. Variances were based on STATA’s internal methods which combines the variance of the samples with the variance of the imputed sets.[3] After MI was performed, 183 subjects with complete covariate information were available in each imputed set, however as explained above, we restricted this study to the 121 mothers with vaginal deliveries. A summary of the imputed values compared to the observed is available in STable 1. The distribution of imputed values did not differ much from the observed (STable 1). The results from the adjusted analysis with only complete cases (Table 3) were substantially different from the results where all 121 mothers were included by imputing values for missing information. Many preterm delivery mothers were less likely to complete and/or return questionnaires, which means that responses were missing more often in the preterm than in the term group. Given that the distributions of the imputed and observed values were quite similar; it could indicate that the MI analysis reduced selection bias in the current study.
