# Supplementary material for: Gut microbiome of mothers delivering prematurely shows reduced diversity and lower relative abundance of Bifidobacterium and Streptococcus
Source: PLoS One. 2017 Oct 25;12(10):e0184336. doi: 10.1371/journal.pone.0184336 (PMC5656300; doi:10.1371/journal.pone.0184336)
Supplement: S2 File — (DOCX) [file pone.0184336.s002.docx]

### Supporting information

### Quality control and sensitivity

Pregibon (1981) Delta-Beta influence statistic was used to identify possible extreme observations in the adjusted logistic models and extreme observations were verified in the original reports. In a sensitivity analysis we included mothers with a spontaneous start of labor with premature rupture of membranes (PROM), even if they had a subsequent caesarian delivery. In the same analysis we also excluded deliveries that were medically induced (i.e. by amniocentesis, prostaglandin and oxytocine), giving a total of 111 deliveries, 94 term, 17 preterm (SFig 1b). In a second sensitivity analysis we compared the diversity of mothers having a caesarian delivery to the women giving birth vaginally. This was to explore the possibility of reverse causation, i.e. whether the short gestation in itself could be a cause of low diversity in the mothers and not the other way around. In that case, the diversity in women giving birth prematurely should be comparable, regardless of delivery mode. In a third sensitivity analysis, we included the women that were given antibiotics on or after the day of labor (n term=9, n preterm=13). We also compared the adjusted analysis using MI to the results from a complete case analysis.

There were no extreme observations in alpha diversity after adjusting for the covariates in the logistic models. There was, however, one influential observation with high abundance of Proteobacteria. The observation was removed from subsequent analyses. In the first sensitivity analysis, when including mothers with PROM and excluding medically induced deliveries (i.e. amniocentesis, prostaglandin and oxytocin), the association between preterm delivery and alpha diversity did not change (Table 3). In the second sensitivity analysis, mothers who had a premature caesarean delivery did not have the same low diversity as mothers delivering vaginally (SFig 5), indicating that short gestation is unlikely to be the reason for low diversity in these mothers. In the third sensitivity analysis, when including mothers who were given antibiotics on or after the day of labor, the associations remained unchanged, but the effect estimates became more significant.
