# Supplementary material for: Gut microbiome of mothers delivering prematurely shows reduced diversity and lower relative abundance of Bifidobacterium and Streptococcus
Source: PLoS One. 2017 Oct 25;12(10):e0184336. doi: 10.1371/journal.pone.0184336 (PMC5656300; doi:10.1371/journal.pone.0184336)
Supplement: S1 Table — Complete case (CC) versus Multiple Imputed (MI) per variable. In total 64 complete cases. NoMIC study. (DOCX) [file pone.0184336.s003.docx]

**S1 Table. Summary of variables with missing values. Complete case (CC) versus Multiple Imputed (MI) per variable. In total 64 complete cases. NoMIC study**

| Variable | N missing (imputed) |  | CC  mean (range) | MI mean (range) |
| --- | --- | --- | --- | --- |
| Income (Euro) | 78 |  | 75,000 (1,875- 250,000) | 83,849 (1,875-250,000) |
| Maternal BMI at start of pregnancy | 7 |  | 23.1 (17.7-37.7) | 23.8 (17.7-37.7) |
|  |  | **Category** | **CC (%)** | **MI (%)** |
| Marital status while pregnant | 1 | Living together or single | 58.8 | 58.9 |
|  |  | Married | 41.2 | 41.1 |
| Education | 10 | <12 years | 6.4 | 6.5 |
|  |  | 12 years | 19.1 | 19.1 |
|  |  | >12 years | 74.6 | 74.4 |
| Ethnicity | 9 | Other | 12.6 | 12.4 |
|  |  | Norwegian | 87.3 | 87.6 |
| Pets | 54 | No | 48.8 | 48.3 |
|  |  | Yes | 51.2 | 51.7 |
| Antibiotics in pregnancy | 8 | No | 76.6 | 76.2 |
|  |  | Yes | 23.4 | 23.8 |
| Smoking at the beginning of pregnancy | 44 | No | 90.7 | 90.1 |
|  |  | Yes | 9.4 | 9.9 |
