# Supplementary material for: Gut microbiome of mothers delivering prematurely shows reduced diversity and lower relative abundance of Bifidobacterium and Streptococcus
Source: PLoS One. 2017 Oct 25;12(10):e0184336. doi: 10.1371/journal.pone.0184336 (PMC5656300; doi:10.1371/journal.pone.0184336)
Supplement: S2 Table — (DOCX) [file pone.0184336.s004.docx]

**S2 Table. Reported reasons for antibiotic use in the 22 women excluded due to antibiotic use on or after the day of labor**

| **Cause of antibiotic use** | **Preterm (n=13) ^a^** | **Term (n=9)^a^** |
| --- | --- | --- |
| Urinary tract infection | 2 | 4 |
| Respiratory infections | 2 | 0 |
| Wound infection | 1 | 1 |
| Mastitis | 2 | 3 |
| C-section | 3 | 4 |
| Group B *Streptococcus* | 2 | 0 |
| *Gardnerella* infection | 1 | 0 |
| Infection, unknown cause | 2 | 0 |
| **^a^**Some women reported several causes, i.e. 27 causes are listed for the 22 women excluded | | |
