# Supplementary material for: Gut microbiome of mothers delivering prematurely shows reduced diversity and lower relative abundance of Bifidobacterium and Streptococcus
Source: PLoS One. 2017 Oct 25;12(10):e0184336. doi: 10.1371/journal.pone.0184336 (PMC5656300; doi:10.1371/journal.pone.0184336)
Supplement: S1 Fig — (DOCX) [file pone.0184336.s005.docx]

**S1a Fig. Flowchart for subject inclusion in main analysis**


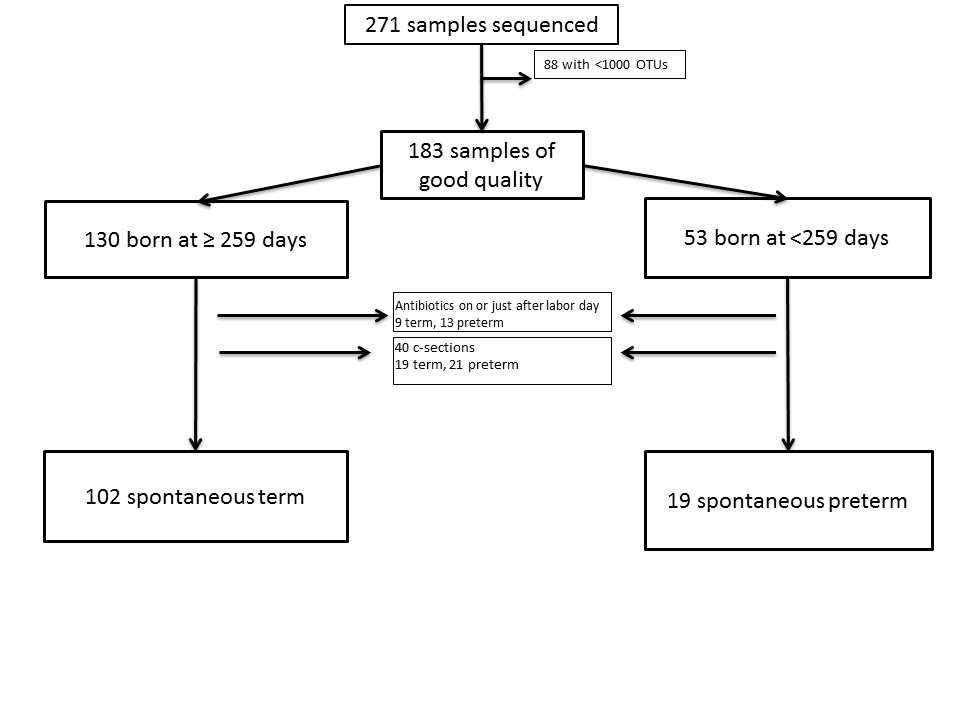


**S1b Fig. Flowchart for subject inclusion in sensitivity analysis**

**
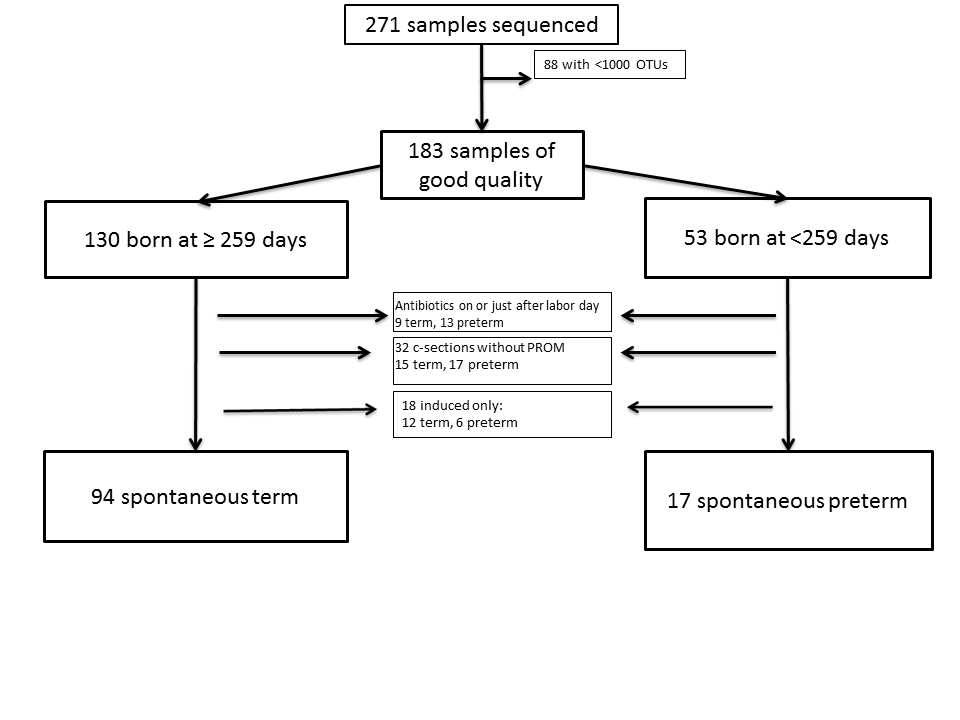
**
