# Supplementary figures and images for: Gut microbiome of mothers delivering prematurely shows reduced diversity and lower relative abundance of Bifidobacterium and Streptococcus
Source: PLoS One. 2017 Oct 25;12(10):e0184336. doi: 10.1371/journal.pone.0184336 (PMC5656300; doi:10.1371/journal.pone.0184336)

**S2 Fig. Directed Acyclic Graph (DAG) for selection of covariates**

**
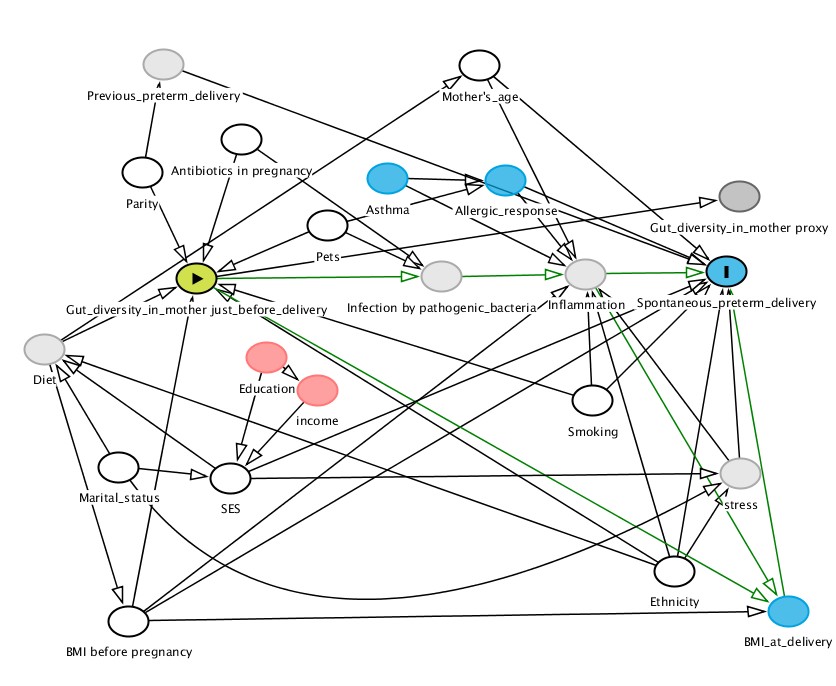
**

Supplement: S2 Fig — (DOCX) [file pone.0184336.s006.docx]
