# Supplementary material for: Gut microbiome of mothers delivering prematurely shows reduced diversity and lower relative abundance of Bifidobacterium and Streptococcus
Source: PLoS One. 2017 Oct 25;12(10):e0184336. doi: 10.1371/journal.pone.0184336 (PMC5656300; doi:10.1371/journal.pone.0184336)
Supplement: S3 Fig — (DOCX) [file pone.0184336.s007.docx]

**S3 Fig. Between communities gut diversity (beta diversity) in unweighted unifrac, visualized with Principal Coordinate Analysis (PCoA).**

**
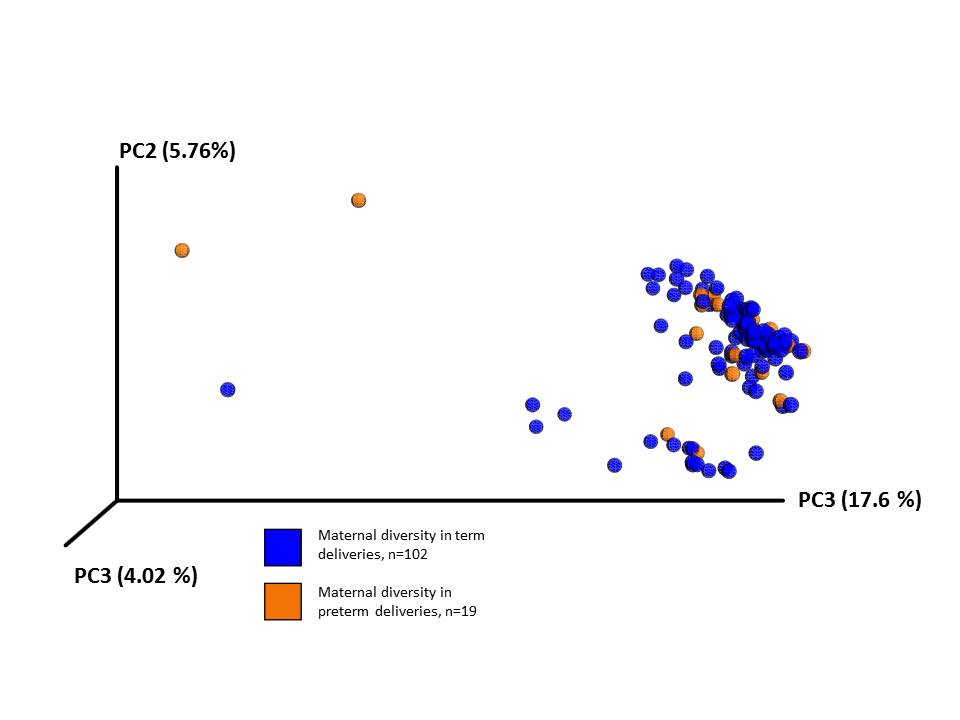
**
