# Supplementary material for: Gut microbiome of mothers delivering prematurely shows reduced diversity and lower relative abundance of Bifidobacterium and Streptococcus
Source: PLoS One. 2017 Oct 25;12(10):e0184336. doi: 10.1371/journal.pone.0184336 (PMC5656300; doi:10.1371/journal.pone.0184336)
Supplement: S4 Fig — Data from 121 mothers, vaginal term delivery (0) and vaginal preterm delivery (1) in the Norwegian Microbiota Study (NoMIC). (DOCX) [file pone.0184336.s008.docx]

**S4 Fig. Box plot comparisons of bacterial families with abundance >0.5% of the total. Data from 121 mothers, vaginal term delivery (0) and vaginal preterm delivery (1) in the Norwegian Microbiota Study (NoMIC)**
