# Supplementary material for: Gut microbiome of mothers delivering prematurely shows reduced diversity and lower relative abundance of Bifidobacterium and Streptococcus
Source: PLoS One. 2017 Oct 25;12(10):e0184336. doi: 10.1371/journal.pone.0184336 (PMC5656300; doi:10.1371/journal.pone.0184336)
Supplement: S5 Fig — Mothers reporting antibiotic use during or after delivery were excluded. (DOCX) [file pone.0184336.s009.docx]

**S5 Fig. Gut diversity (measured by Shannon) in mothers of 4 delivery types: Vaginal term (N=102), C-section term (N=19), C-section preterm (N=21) and vaginal preterm (N=19). Mothers reporting antibiotic use during or after delivery were excluded.**
